# Implementation of the Participatory Approach for Supervisors to Increase Self-Efficacy in Addressing Risk of Sick Leave of Employees: Results of a Cluster-Randomized Controlled Trial

**DOI:** 10.1007/s10926-016-9652-3

**Published:** 2016-07-11

**Authors:** S. M. Ketelaar, F. G. Schaafsma, M. F. Geldof, R. A. Kraaijeveld, C. R. L. Boot, W. S. Shaw, U. Bültmann, J. R. Anema

**Affiliations:** 1 Department of Public and Occupational Health, EMGO+ Institute for Health and Care Research, VU University Medical Center, Amsterdam, The Netherlands; 2 Research Center for Insurance Medicine, Collaboration Between AMC-UMCG-UWV-VUmc, Amsterdam, The Netherlands; 3 Body@Work, Research Center Physical Activity, Work and Health, TNO-VU University Medical Center, Amsterdam, The Netherlands; 4 Liberty Mutual Research Institute for Safety, Hopkinton, MA USA; 5 University of Massachusetts Medical School, Worcester, MA USA; 6 Department of Health Sciences, Community and Occupational Medicine, University Medical Center Groningen, University of Groningen, Groningen, The Netherlands

**Keywords:** Participatory approach, Workplace, Sick leave, Prevention, Supervisors, Randomized controlled trial

## Abstract

*Purpose* To study the effectiveness of a multifaceted strategy to implement the participatory approach (PA) for supervisors to increase their self-efficacy in addressing risk of sick leave of employees. *Methods* Supervisors from three organizations were invited to participate. Randomization was performed at department level. Supervisors (n = 61) in the intervention departments received the implementation strategy consisting of a working group meeting, supervisor training in PA application, and optional supervisor coaching. Supervisors in the control departments (n = 55) received written information on PA. The primary outcome was supervisors’ self-efficacy to apply the PA, measured at baseline and 6 months’ follow-up. The number of employees with whom supervisors discussed work functioning problems or (risk of) sick leave was also assessed. Effects were tested using multilevel analyses. *Results* The strategy did not increase self-efficacy to apply the PA. Subgroup analyses showed that self-efficacy increased for supervisors who at baseline reported to have discussed (risk of) sick leave with less than three employees during the last 6 months (B = 1.42, 95 % CI 0.34–2.50). Furthermore, the implementation strategy increased the number of employees with whom supervisors discussed work functioning problems or risk of sick leave (B = 1.26, 95 % CI 0.04–2.48). *Conclusion* Although the implementation strategy cannot be recommended for all supervisors, for supervisors who less frequently discuss (risk of) sick leave with employees the implementation strategy might be helpful.

*Trial registration* NTR3733.

## Introduction

When an employee has work functioning problems due to health complaints and is at risk of sick leave, employees and their supervisors usually do not find it easy to discuss these problems [1–3]. To prevent sick leave, it is important to act timely and to properly address work functioning problems. Facilitating supervisors and employees to discuss work functioning problems due to health complaints might be helpful.

The Participatory Approach (PA) is effective to improve return-to-work (RTW), to shorten the duration of sick leave [4–8] and to reduce various health complaints of employees [9–11]. It encompasses a workplace intervention protocol, in which supervisors and employees separately identify work functioning problems due to health complaints and subsequently discuss and solve these problems together. In previous studies, the PA was applied to address barriers for RTW of employees on sick leave, guided by an RTW coordinator [6, 7, 9, 10]. In the present study, we take an innovative approach, focusing on the application of the PA to identify and tackle work functioning problems early. Thereby, we aim to prevent employees from sick leave, thus using PA as indicated prevention targeting employees with early symptoms of being at risk of sick leave [12].

To date, the PA was applied by an occupational health professional (OHP) as RTW coordinator, acting as process leader. However, supervisors are arguably the first, together with colleagues, to notice that an employee has work functioning problems or is at risk of sick leave. Moreover, the supervisor is considered a key factor in managing and optimizing work functioning of an employee with health problems, and in providing the necessary conditions to help the employee to remain at work [1, 13, 14]. When applying the PA as a preventive strategy, it seems appropriate that the supervisor applies the PA instead of an OHP, thus acting as both a process leader and as a participant in joint-problem solving together with the employee.

Several barriers may impede implementation of the PA within an organization [15–17]. At the organizational level, the PA might not comply with organizational sick-leave policies and practices. At the level of supervisors, barriers may be lack of self-efficacy to discuss work functioning problems with employees with health complaints and to jointly solve these problems, lack of the required attitude, and lack of sufficient knowledge about health complaints, the possibilities of work adaptations for employees with health complaints, and when to consult an OHP [15–17]. These barriers correspond with the Attitude-Social Influence-Self-efficacy (ASE) model [18]. The ASE model assumes that behavior (in this case the supervisor discussing work functioning problems and risk of sick leave with the employee) can be predicted by the intention to perform that behavior, which is in turn determined by an individual’s attitude, social influence from others, and self-efficacy to perform that behavior [18]. Furthermore, at the employee level, employees may experience a lack of empathy, respect and support from their supervisor. In addition, employees may experience that their supervisor do not provide sufficient possibilities for joint problem-solving regarding work functioning problems. To enable supervisors to effectively apply the PA to prevent sick leave of employees, a multifaceted implementation strategy is needed. Organizational barriers should be tackled by involving relevant stakeholders and jointly investigating the main challenges in achieving the change of practice within the specific organizational context, and selecting appropriate strategies and measures at different organizational levels [19]. Our multifaceted implementation strategy consisted of three elements [20]; (1) a working group meeting in each participating organization with relevant stakeholders; (2) a half-day training for supervisors; and (3) the possibility for supervisors to receive individual coaching in application of the PA.

Because supervisors find it difficult to discuss work functioning problems and risk of sick leave with employees [1–3], the main objective of our study was to investigate the effectiveness of the multifaceted implementation strategy of the PA on supervisors’ self-efficacy to apply the PA at 6-months’ follow-up. Secondary outcomes were supervisors’ attitude, social influence and intention to apply the PA, supervisors’ application of the PA, and the percentage and sick-leave duration of sick-listed employees.

## Methods

### Study Design

In a cluster-randomized controlled trial the multifaceted implementation strategy (intervention) was compared with a minimal implementation strategy (control). The protocol was published previously [20]. Three organizations now referred to as study sites participated in the study: a steel factory, a university medical center, and a university. Random allocation to either the intervention group or the control group was performed at department level to limit contamination between supervisors in both groups. To keep differences between the intervention group and the control group as small as possible, we repeatedly took two departments within a study site that were similar regarding the number of participating supervisors within the departments and the departments’ sick-leave frequencies. We then randomly assigned one of these two departments to the intervention group, and the other to the control group. In case of very small numbers of participating supervisors from one department, these were combined with another department with similar sick-leave frequencies to achieve equal numbers of supervisors in both groups. Randomization was performed by an independent researcher who was not involved in the study. Researchers, supervisors, managers, human resource professionals (HRPs), and occupational health professionals (OHPs) were not blinded to the intervention. The study was performed in 2012 and 2013, outcome measurement took place at baseline and after 6 months. The study protocol was approved by the Medical Ethics Committee of the VU University Medical Center, Amsterdam, the Netherlands. The report of this study follows the Consolidated Standards of Reporting Trials guidelines [20].

### Study Participants

The participating study sites employed about 20,000 employees, of whom 1,400 had a supervisory role for 10 employees or more. Based on earlier implementation studies, each working group aimed to include six stakeholders: supervisors, employees, managers at department level, HRPs, OHPs, and occupational physicians (OPs) [16, 21]. The contact person within each study site suggested stakeholder representatives, who were approached by the researchers to participate in the working group.

Supervisors were eligible for participation in the PA application training if they were at least 18 years old and worked at least 24 h per week. The inclusion criterion of supervising at least 10 employees, as specified in the study protocol [20], was dropped because this would have led to too few participating supervisors. Supervisors whose contracts would end within 1 year after baseline, and supervisors who were not able to fill out questionnaires in the Dutch language were excluded. Supervisors were initially not directly approached for participation. The study site (mostly the department managers in collaboration with HR advisors) first made an inventory of supervisors who might be eligible and interested in the training. The decision about eligibility and interest of the supervisor was dependent on the views of the department managers and HR advisors of the study site. In some cases they simply forwarded an email with information about the supervisor training to all supervisors in a department, and in other cases they sent an invitation to a small group of supervisors. These supervisors were then approached by the research team and invited to participate.

### Intervention

#### Multifaceted Implementation Strategy

The multifaceted implementation strategy was applied in the intervention group and consisted of three components, following the baseline measurement (month 1): one working group meeting per study site with stakeholder representatives (month 2), supervisor training in application of the PA (months 3), and optional supervisor coaching (month 4–12) [20].

##### Working Group Meeting

In each study site, one 2-hour working group meeting, chaired by an in-company OHP, was organized. In this meeting, participating stakeholder representatives discussed signals of work functioning problems, situations in which supervisors should apply the PA, and barriers to and facilitators for PA implementation within the specific study site. The results from this working group meeting were summarized in a manual for the supervisor training and coaching. As such, a customized training manual was developed for each study site.

##### Supervisor Training

Supervisors were invited to participate in a 4-hour training in PA application, and an optional 2-hour follow-up training. The training was provided by in-company OHPs, who were trained by the researchers (RAK, FGS). The training included how to identify an employee with work functioning problems or at risk of sick leave, how to discuss the risk of sick leave with the employee, the steps within the protocol on PA application, and how to apply the protocol in daily practice. The training was partly based on the supervisor training by Shaw et al. [8], and included an oral presentation, group discussions, and role-playing to practice application of the PA protocol.

The protocol on PA application consisted of seven steps to identify and solve employees’ work functioning problems due to health complaints (Box 1). There is no fixed timeline for these seven steps planned in advance, as it depends on the work-related issue, the availability of both persons, and of the expected duration to implement any work adjustment. The PA protocol was primarily targeted towards employees with work functioning problems due to health conditions who are at risk of sick leave. In practice, there is no difference in guidance of sick-listed and non-sick-listed employees with work functioning problems. Therefore, supervisors were also instructed to apply the protocol to sick-listed employees, i.e. to jointly identify and solve barriers to RTW. Within the PA application, the supervisor acts as both participant (i.e. the supervisory role) and process leader. However, if needed, the supervisor or the employee could ask an OHP to act as process leader.

**Box 1:** Protocol for application of PA

Meeting 1	Step 1	Supervisor addresses the employee’s work functioning problems due to health complaints or risk of sick leave and informs the employee about the PA protocol
Preparation	Step 2	Employee makes an inventory of his or her work tasks and activities, prioritizes work functioning problems regarding these activities, and thinks of possible solutions for the two most important work functioning problems
	Step 3	Supervisor makes an inventory of the employee’s work tasks and activities, prioritizes work functioning problems regarding these activities, and thinks of possible solutions for the two most important work functioning problems
Meeting 2	Step 4	Supervisor and employee discuss work functioning problems and possible solutions, and assess the applicability of these solutions
	Step 5	Supervisor and employee agree on an action plan to realize solutions
Realisation	Step 6	Solutions are prepared and realized
Meeting 3	Step 7	Supervisor and employee evaluate the action plan and the realized solutions

##### Supervisor Coaching

Throughout the study period, coaching by an in-company OHP was available for all supervisors in the intervention group when the supervisor or the employee expected problems during the PA application. For example, a supervisor could ask the OHP to help prepare an up-coming meeting with an employee or to guide the actual PA application by functioning as a process leader during a supervisor-employee meeting.

##### Minimal Implementation Strategy

The minimal implementation strategy in the control group consisted of the distribution of written information on PA. After completion of the study, departments in the control group were offered to receive the multifaceted implementation strategy.

### Outcomes

All outcome measures were obtained from participating supervisors at baseline and at 6 months’ follow-up.

#### Primary Outcome

The primary outcome was supervisors’ self-efficacy regarding joint problem-solving (i.e. applying the PA) to improve work functioning of employees with health problems and to prevent sick leave of these employees. To measure self-efficacy, three items of the competence scale of Spreitzer and colleagues’ Empowerment questionnaire were modified to fit the context of this study [22] (Cronbach’s alpha = 0.77). An example item is “I am confident about my ability to think of and realize solutions together with my employee”. The response could be provided on a seven-point Likert scale from 1 (totally disagree) to 7 (totally agree) [22]. A summary score was calculated ranging from 3 to 21.

#### Secondary Outcomes

Attitude and social influence were assessed regarding joint problem-solving to improve the work functioning of employees with health problems and to prevent sick leave. Furthermore, intention to apply joint problem-solving was assessed. Response categories for all items ranged from 1 (totally disagree) to 5 (totally agree). Three items were used to assess attitude (range of sum score 3–15; Cronbach’s alpha = 0.56), for example “To discuss and solve these situations is important for me”. To assess social influence, two items were used:”My organization encourages me to engage in joint problem-solving with an employee” and “Employees expect me to think of and realize solutions together”. Because the Cronbach’s alpha for these combined items was low (0.10), it was decided to report results for the two items for social influence separately (each with score range 1–5). Intention to apply joint problem-solving was assessed with one item (score range 1–5): “It is very likely that my employee and I would think of and realize solutions together”.

In addition, supervisors’ self-efficacy to discuss work functioning problems or the risk of sick leave with the employee was assessed with three self-formulated items (Cronbach’s alpha = 0.83). For example “I am confident about discussing these situations with my employee”. The response categories ranged from 1 (totally disagree) to 5 (totally agree), leading to a sum score with a range of 3–15.

Lastly, supervisors were asked how many employees they supervised in total, how many of their employees were sick-listed due to health complaints in the last 6 months, and how many calendar days in total these employees were sick-listed in the last 6 months. The percentage of sick-listed employees and the average duration of sick leave in calendar days were calculated per supervisor.

As an implementation indicator the actual application of the PA by the supervisor was assessed, by asking supervisors with how many employees they had discussed work functioning problems or sick leave during the last 6 months.

#### Possible Confounders and Effect Modifiers

Several factors were taken into account as possible confounders or effect modifiers based on the available literature [7–9]. Supervisors’ age, sex, study site, number of employees under their supervision, and number of years of supervisor experience were assessed. In addition, the number of employees at risk of sick leave and the number of sick-listed employees at baseline were taken into account. Lastly, the number of employees with whom supervisors had discussed (risk of) sick leave in the last 6 months, as reported at baseline, was considered as possible experience indicator and as potential confounder or effect modifier.

### Sample Size

Based on previous research, the multifaceted implementation strategy of the PA was expected to increase supervisors’ self-efficacy regarding joint problem-solving (i.e. applying the PA) to improve the work functioning of employees with health problems and to prevent sick leave of these employees [17]. To adjust for possible effects due to cluster-randomization at department level, an intra-class correlation coefficient (ICC) of 0.05 was used. Taking into account a mean score of 6.02 and SD of 0.88 on the competence scale of Spreitzer and colleagues [22], a power (1-beta) of 0.80 and an alpha of 0.05 (two-tailed) and assuming a drop-out rate of 20 %, a total sample size of 107 supervisors was required to detect a 10 % increase in self-efficacy.

### Statistical Analyses

Intention-to-treat analyses were performed at the supervisor level. Baseline characteristics were calculated using descriptive statistics. A drop-out analysis was performed to determine whether non-completers and completers (i.e. those who filled out baseline and 6 months’ follow-up questionnaires and those who did not) differed in the primary outcome at baseline, using a Mann–Whitney U test. Multilevel analyses were performed for all outcome variables with the supervisor clustered within the department. We only used complete cases for the analyses; all cases were adjusted for the baseline value of the particular outcome.. Supervisors were analyzed as a total group, as OHP were only consulted three times. Both crude and adjusted analyses (adjusted for sex of the supervisor, years of supervisory experience, and the number of employees at risk of sick leave at baseline) were performed. Per-protocol analyses were performed with a nominal variable (1 = control group, 2 = intervention group and received training, 3 = intervention group but did not receive training) as the independent variable for the analyses. Lastly, effect modification was investigated for the possible effect modifiers (supervisors’ age, sex, study site, number of employees under their supervision, and number of years of supervisor experience) using a *p* value <0.1 of the interaction term to indicate relevant effect modification.

In case of effect modification, stratified post hoc analyses were also performed. The statistical significance level was set at α = 0.05. All multilevel analyses were performed using MLwiN; all other analyses were performed using SPSS 20.0 (IBM Corp, Released 2011, IBM SPSS Statistics for Windows, Version 20.0. Armonk, NY).

## Results

### Flow of Study Participants

**Fig. 1:**
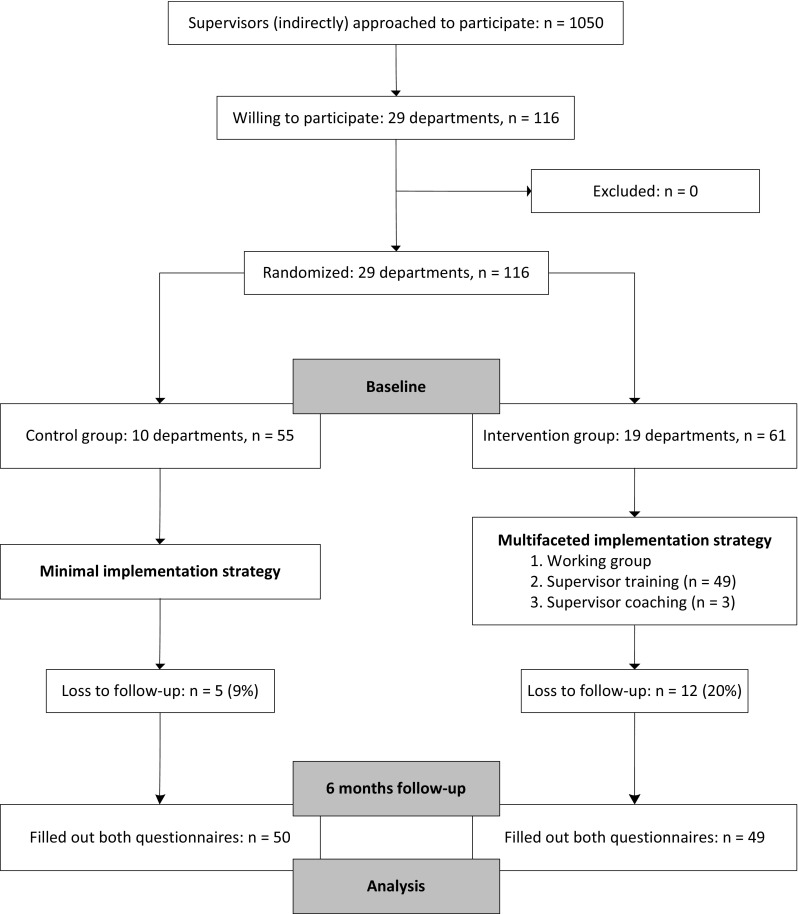
Participant flow

Approximately 1050 supervisors were approached by their department manager for participation (Fig. 1). In total, 116 supervisors (11 %) working in 29 departments were willing to participate and met the inclusion criteria. Ten departments with 55 participating supervisors were randomly assigned to the control group and 19 departments with 61 participating supervisors to the intervention group. Eighty percent of supervisors in the intervention group (n = 49) participated in the training. Non-participation in the training was mostly due to working shifts and therefore not being able to attend the training. Three supervisors (5 %) in the intervention group requested one coaching session. In total, 50 supervisors in the control group (91 %) and 49 supervisors in the intervention group (80 %) filled out both questionnaires and were included in the analyses. The drop-out analysis showed that there were no significant differences between completers and non-completers (p = 0.37) on self-efficacy as primary outcome.

### Baseline Characteristics

As shown in Table 1, the largest proportion of participating supervisors was employed by the steel factory and no university supervisors took part in the control group. The majority of participating supervisors were male and the average supervisory experience was approximately 10 years. The majority of the supervisors (64 %) considered their main job description to be managerial. Other job descriptions were technical (11 %), (para) medical (10 %), or other such as research, education or administrative (10 %). The job descriptions of the supervised workers varied greatly depending on the type of organization they worked, such as technical (26 %), (para)medical (38 %), administrative (12 %), research or educational (9 %), managerial (8 %). Sick-leave rates in the year before the trial were 4.6 % for the steel factory, 3.5 % for the university medical center, and 4.7 % for the university. About one in seven supervisors were familiar with the PA.

**Table 1:** Baseline characteristics of the ‘supervisor’ study population (n = 116)

	Intervention group (n = 61)		Control group (n = 55)	
Study site				
Steel factory, n (%)	29	(48 %)	33	(60 %)
University medical centre, n (%)	22	(36 %)	22	(40 %)
University, n (%)	10	(16 %)	0	(0 %)
Male sex, n (%)	35	(57 %)	36	(66 %)
Age in years, M (SD)	47	(7)	46	(8)
High level of education (higher professional education or university), n (%)	47	(77 %)	38	(69 %)
Supervisory experience in years, M (SD)	10	(7)	9	(7)
Number of supervised employees, M (SD)	28	(22)	27	(27)
Familiar with PA; yes, n (%)	8	(13 %)	9	(16 %)
Applied PA in last 6 months; yes, n (%)	4	(7 %)	1	(2 %)

### Self-Efficacy Regarding PA Application (Primary Outcome)

As shown in Table 2, self-efficacy regarding joint problem-solving at baseline was relatively high in both groups. There was no significant difference over time between the groups in both the crude and adjusted analyses. We analyzed whether there was any difference in effect due to the study site, this was not the case.

**Table 2:** Mean scores on outcomes at baseline and 6 months’ follow-up and multilevel analysis results

	Intervention group (n = 49)		Control group (n = 50)		ML model crude			ML model adjusted^a^		
	M	(SD)	M	(SD)	B	(SE)	[95 % CI]	B	(SE)	[95 % CI]
Primary outcome										
Self-efficacy regarding joint problem-solving (range 3–21)										
Baseline	16.3	(2.2)	17.0	(1.4)						
6 months’ follow-up	17.2	(2.3)	16.6	(2.2)	0.68	(0.58)	[−0.46 to 1.82]	0.54	(0.62)	[−0.68 to 1.76]
*Secondary outcomes*										
Attitude regarding joint problem-solving (range 3–15)										
Baseline	12.9	(1.1)	12.8	(1.2)						
6 months’ follow-up	12.8	(1.1)	13.1	(1.2)	−0.36	(0.22)	[−0.79 to 0.07]	−0.38	(0.23)	[−0.83 to 0.07]
Social influence from organization regarding joint problem-solving (range 1–5)										
Baseline	3.4	(0.7)	3.7	(0.7)						
6 months’ follow-up	3.6	(0.8)	3.8	(0.7)	−0.12	(0.15)	[−0.41 to 0.17]	−0.17	(0.15)	[−0.46 to 0.12]
Social influence from employees regarding joint problem-solving (range 1–5)										
Baseline	3.7	(0.7)	3.7	(0.6)						
6 months’ follow-up	3.7	(0.8)	3.8	(0.6)	−0.04	(0.13)	[−0.29 to 0.21]	−0.08	(0.14)	[−0.35 to 0.19]
Intention to apply joint problem-solving (range 1–5)										
Baseline	4.2	(0.6)	4.1	(0.4)						
6 months’ follow-up	4.1	(0.5)	4.1	(0.4)	−0.01	(0.09)	[−0.19 to 0.17]	−0.05	(0.09)	[−0.23 to 0.13]
Self-efficacy to discuss work functioning problems or (risk of) sick leave (range 3–15)										
Baseline	10.5	(2.1)	10.5	(1.8)						
6 months’ follow-up	11.3	(2.1)	10.9	(1.9)	0.39	(0.35)	[−0.30 to 1.08]	0.29	(0.36)	[−0.42 to 1.00]
Percentage of employees who were sick-listed in last 6 months										
Baseline	0.25	(0.20)	0.33	(0.26)						
6 months’ follow-up	0.19	(0.19)	0.29	(0.29)	−0.04	(0.04)	[−0.12 to 0.04]	−0.02	(0.04)	[−0.10 to 0.06]
Average duration of sick-leave (calendar days) in last 6 months										
Baseline	2.8	(3.1)	4.2	(4.4)						
6 months’ follow-up	4.4	(6.9)	3.6	(4.9)	1.00	(1.40)	[−1.74 to 3.74]	1.99	(1.16)	[−0.28 to 4.26]
Number of employees with whom work functioning problems or risk of sick leave was discussed in last 6 months										
Baseline	1.0	(1.6)	1.2	(1.5)						
6 months’ follow-up	2.0	(3.9)	0.8	(1.2)	**1.28**	**(0.60)**	**[0.10**–**2.46]**	**1.26**	**(0.62)**	**[0.04**–**2.48]**
Number of employees with whom sick-leave was discussed in last 6 months										
Baseline	1.7	(1.5)	1.7	(2.2)						
6 months’ follow-up	1.8	(2.6)	1.3	(1.3)	0.49	(0.44)	[−0.37 to 1.35]	0.50	(0.45)	[−0.38 to 1.38]

Bold values are statistically significant as 95 % confidence interval does not encompass zero

^a^Confounders: years of supervisory experience, number of employees at risk of sick leave at baseline, and supervisor’s sex

A subsequent per-protocol analysis showed that self-efficacy regarding joint problem-solving of the subgroup of supervisors in the intervention group that had followed the training (N = 41) had increased over time (16.1–17.3), while it had decreased (17.0–16.6) in the subgroup that had not followed the training (N = 8). However, the subgroup that had followed the training showed no significant difference over time compared to the control group.

The number of employees with whom the supervisor had discussed (risk of) sick leave during the last 6 months, measured at baseline, was a significant effect modifier of self-efficacy regarding joint problem-solving. For the stratified analysis, the median was used as cut-off point to differentiate between a low number of employees (0–2) and a high number of employees (≥3) with whom the supervisor had discussed these issues. Results of the stratified analysis of self-efficacy regarding joint problem-solving are shown in Table 3. When looking at the subgroup of supervisors who (at baseline) had discussed (risk of) sick-leave with 0–2 employees, the control group showed a slightly larger decrease in self-efficacy when compared to the whole group of supervisors in the control group (which is shown in Table 2). In the intervention group, this subgroup showed a slightly larger increase than the whole intervention group. The difference between the control group and intervention group regarding supervisors who had discussed (risk of) sick-leave with 0–2 employees was statistically significant (B = 1.42, 95 % CI 0.34–2.50). This difference was not found in the subgroup of supervisors who (at baseline) had discussed (risk of) sick-leave with three or more employees.

**Table 3:** Results of stratified analyses according to the number of employees with whom supervisors had discussed the (risk of) sick-leave during the last 6 months, measured at baseline

	Intervention group		Control group		ML model crude			ML model adjusted^a^		
	M	(SD)	M	(SD)	B	(SE)	[95 % CI]	B	(SE)	[95 % CI]
Self-efficacy regarding joint problem-solving (range 3–21)										
Discussed (risk of) sick leave with 0–2 employees in last 6 months^b^										
Baseline	16.2	(2.4)	17.0	(1.4)						
6 months’ follow-up	17.4	(2.3)	16.2	(2.0)	**1.40**	**(0.55)**	**[0.32**–**2.48]**	**1.42**	**(0.55)**	**[0.34**–**2.50]**
Discussed (risk of) sick leave with ≥3 employees in last 6 months^b^										
Baseline	16.5	(2.0)	17.1	(1.4)						
6 months’ follow-up	16.9	(2.4)	17.1	(2.6)	0.03	(0.88)	[−1.69 to 1.75]	−1.02	(0.78)	[−2.55 to 0.51]

Bold values are statistically significant as 95 % confidence interval does not encompass zero

^a^Confounders: years of supervisory experience, number of employees at risk of sick leave at baseline, and supervisor’s sex

^b^Measured at baseline

### Secondary Outcomes

As shown in Table 2, the mean score on attitude towards joint problem-solving was relatively high at baseline and remained almost the same over time in both groups, with no significant difference over time between the groups. The same pattern was seen regarding social influence towards joint problem-solving and intention to apply joint problem-solving. Self-efficacy to discuss work functioning problems or risk of sick leave with employees increased in both groups, with no significant difference over time between the groups.

The percentage of sick-listed employees per supervisor decreased over time in both groups, with no significant difference over time between the groups. The average sick-leave duration decreased in the control group while it increased in the intervention group, but this difference was not statistically significant.

The number of employees with whom the supervisor discussed work functioning problems or risk of sick leave in the last 6 months decreased in the control group and increased in the intervention group. This difference over time between the groups was statistically significant (B = 1.26, 95 % CI 0.04–2.48). The number of employees with whom supervisors discussed actual sick leave also decreased in the control group but remained fairly similar in the intervention group, with no significant difference over time between the groups.

## Discussion

Our primary outcome was the effectiveness of a multifaceted implementation strategy of the PA on supervisors’ self-efficacy to apply the PA. Comparing this multifaceted implementation strategy with a minimal strategy, we found that it did not significantly increase supervisors’ self-efficacy to apply the PA, i.e. to discuss employees’ work functioning problems and to engage in joint problem-solving improve work functioning and prevent sick leave. Subgroup analyses showed that self-efficacy increased for supervisors who at baseline reported to have discussed (risk of) sick leave with less than three employees during the last 6 months. The effectiveness of the implementation strategy was further measured by the actual PA application as an implementation indicator. The multifaceted implementation strategy increased the average number of employees with whom supervisors discussed work functioning problems or risk of sick leave, when compared to the control group. Regarding all other outcomes, no statistically significant differences over time were found between the group who were targeted with the multifaceted implementation strategy and the control group.

### Interpretation of Findings

In this study, we investigated a multifaceted strategy to implement PA application to prevent sick leave of employees. Two innovative elements were introduced: using PA not only for sick-listed employees, but also for employees with work functioning problems or at risk of sick leave due to health complaints; and supervisors applying the PA and thus acting as both participant (i.e. the supervisory role) and process leader (instead of an OHP acting as process leader).

According to the ASE model, behavior can be predicted by the intention to perform that behavior, which is in turn determined by the individual’s attitude, social influence from others, and self-efficacy to perform that behavior [18]. Our implementation indicator showed that the implementation strategy had an effect on the actual behavior performance of the supervisor, i.e. on the application of the PA. This effect was only found regarding PA application for employees at risk of sick leave, and not for employees who were already sick-listed. It is not surprising that the implementation strategy has not increased supervisors’ application of the PA for sick-listed employees: this was already part of their practice. It was particularly important to increase discussing work functioning problems and engaging in joint problem-solving earlier, before the employee goes on sick leave. The implementation strategy did indeed show this effect. However, the number of times that the supervisors discussed work functioning problems or risk of sick leave was still not very high at follow-up. Although the training session did pay attention to how to identify risk of sick leave, supervisors might still find this aspect difficult.

Because supervisors may be insecure to discuss work functioning problems and risk of sick leave with employees [1], our multifaceted implementation strategy was primarily aimed towards increasing supervisors’ self-efficacy in addressing these issues and engaging in joint problem-solving with the employee. Our post hoc analyses showed that the strategy was only effective in increasing self-efficacy of supervisors who had less recent experience in discussing (risk of) sick leave with their employees. Although these findings need to be replicated in future research, it seems that regarding self-efficacy, the implementation strategy is only useful for less experienced supervisors, and perhaps also for supervisors who are experienced supervisors but find it difficult to perform this specific supervisory task. Therefore, it might be valuable to include training in PA application in leadership training programs. However, it should be noted that the clinical relevance of the effect in our study is questionable: the increase in self-efficacy remains quite small. In addition, supervisors in the participating study sites already talked with their employees on a regular basis. The multifaceted strategy to implement the PA might be more effective in companies in which supervisors are less familiar with their role as case manager regarding (prevention of) sick leave. It should also be taken into account that there was a higher drop-out rate in the intervention group than in the control group. We performed a drop-out analysis which showed that there were no baseline differences regarding the primary outcome between supervisors who did and supervisors who did not drop out. However, it cannot be ruled out that the supervisors who dropped out would have had lower scores on the primary outcome at 6 months’ follow-up.

Another factor to take into account is that our underlying framework of data collection around the concept of supervisors’ self-efficacy suggests that supervisor behavior is largely mediated by mastery of communication skills that can be trained. However, other forces such as incentives, peer recognition, normative beliefs, senior management commitment or operational pressures may also influence participatory behavior of supervisors. This aspect could be better explored in future work.

Although the actual performance of the desired behavior increased, the aspects that are thought to predict behavior performance did not. Possibly, there was too little room for improvement, because baseline scores for attitude, social influence and intention regarding joint problem-solving were high. Another explanation might be that the assessment method of these aspects was insufficiently capable of measuring difference over time. The ASE aspects were measured using self-formulated items and were thus not validated regarding responsiveness. In addition, the Cronbach’s alpha of attitude was fairly low (0.56), indicating that the scale might have been insufficiently reliable.

Ultimately, PA application is aimed towards preventing sick leave. However, our study showed no statistically significant effect of the implementation strategy on the percentage of sick-listed employees due to health complaints and on sick-leave duration. Our study showed that the number of times that the PA was applied in cases with risk of sick leave increased over time. However, this does not mean that supervisors’ application of the PA reduces the number of sick-listed employees. It can be hypothesized that discussing the risk of sick leave leads to temporary part-time sick leave as one of the solutions resulting from the application of the participatory approach. Temporary part-time sick leave may be deemed necessary by the supervisor and employee to keep a remaining healthy work functioning as much as possible. Furthermore, PA application also requires competence from the employee in analyzing their specific work functioning problems. The process evaluation of our study showed that one of the reasons for supervisors to not apply the PA was that it was too difficult for employees [23].

### Methodological Considerations

Several methodological aspects should be considered. First of all, our method of recruiting supervisors for participation may have led to selection bias. Department managers and HR advisors were asked to make an inventory of supervisors who might be interested in the training and these supervisors were then approached for participation. Arguably, voluntary participation always leads to some form of selection bias, but in this case an additional selection was made by the study site. This did not lead to a difference between the intervention and the control group, because randomization took place after agreement to participate. However, it may have accounted for the relatively high baseline scores on participating supervisors’ attitude, social influence and intention regarding joint problem-solving. Next, there is the chance of recall bias for the supervisors in the intervention group. Although, all participating supervisors were well aware of the objective of this trial, and received similar questionnaires related to their employees with health related work problems. Another issue worth mentioning is the small risk of contamination between supervisors from different departments and between groups.

Next, our primary outcome was measured using a selection of items from the competence scale of Spreitzer and colleagues’ Empowerment questionnaire [22], which were modified to fit the study context. The internal consistency of our scale was sufficient, however it has not been validated regarding responsiveness to change over time. The secondary outcomes related to the ASE model were not validated in a supervisor population, and due to multiple testing for these outcomes, the risk of a Type 1 error cannot be excluded. Both issues are considered a limitation of this study [18].

Furthermore, our method of measuring the percentage of sick-listed employees and sick-leave duration might have been inaccurate. These outcomes were assessed by asking supervisors to report how many of their employees were sick-listed during the last 6 months, and how many calendar days these employees were sick-listed in total. As these questions are difficult to answer for a team of employees, recall bias cannot be excluded. Objective sick-leave data might have provided more exact data. Unfortunately, obtaining objective data for each supervisor was not possible, because these data were not all recorded at the supervisor level. Lastly, the number of discussions with employees as an implementation indicator needs to be interpreted with caution, as this was calculated as a total number of discussions for all employees for which recall bias cannot be excluded. Moreover, the actual number of employees in need for a discussion during the follow up period has to be taken into account.

## Conclusion

The multifaceted strategy to implement the PA did not increase self-efficacy to apply the PA. For supervisors who less frequently discuss (risk of) sick leave with employees the implementation strategy might be helpful. Furthermore, the implementation strategy increased the number of times that supervisors discussed work functioning problems or risk of sick leave to prevent sick leave. The implementation strategy may be recommended for supervisors who less frequently discuss (risk of) sick leave with employees.
